# Inferring gene-to-phenotype and gene-to-disease relationships at Mouse Genome Informatics: challenges and solutions

**DOI:** 10.1186/s13326-016-0054-4

**Published:** 2016-05-20

**Authors:** Susan M. Bello, Janan T. Eppig, the MGI Software Group

**Affiliations:** Mouse Genome Informatics, The Jackson Laboratory, Bar Harbor, ME 04609 USA

**Keywords:** Phenotype, Genotype, Disease, Mouse, Annotation

## Abstract

**Background:**

Inferring gene-to-phenotype and gene-to-human disease model relationships from annotated mouse phenotypes and disease associations is critical when researching gene function and identifying candidate disease genes. Filtering the various kinds of genotypes to determine which phenotypes are caused by a mutation in a particular gene can be a laborious and time-consuming process.

**Methods:**

At Mouse Genome Informatics (MGI, www.informatics.jax.org), we have developed a gene annotation derivation algorithm that computes gene-to-phenotype and gene-to-disease annotations from our existing corpus of annotations to genotypes. This algorithm differentiates between simple genotypes with causative mutations in a single gene and more complex genotypes where mutations in multiple genes may contribute to the phenotype. As part of the process, alleles functioning as tools (e.g., reporters, recombinases) are filtered out.

**Results:**

Using this algorithm derived gene-to-phenotype and gene-to-disease annotations were created for 16,000 and 2100 mouse markers, respectively, starting from over 57,900 and 4800 genotypes with at least one phenotype and disease annotation, respectively.

**Conclusions:**

Implementation of this algorithm provides consistent and accurate gene annotations across MGI and provides a vital time-savings relative to manual annotation by curators.

## Background

Genetic mutations in mouse models have proven a valuable tool in investigating gene function and facilitating research into human disease. The phenotypes associated with these mutations in mice occur in the context of other defined or undefined mutations in their genome. To determine if a phenotype is caused by a mutation in a specific gene, providing insight into gene function, the impact of each allele in the genotype needs to be evaluated. Doing this manually is a laborious and time-consuming process. Intensely researched genes may have dozens of alleles each with multiple genotypes. The mouse gene *Pax6* (MGI:97490) alone has 53 mutant alleles present in some 150 mouse genotypes with phenotype annotations in Mouse Genome Informatics (MGI, as of 12/29/2015). Only a fraction of these reported phenotypes are caused solely by the mutation(s) in *Pax6*.

MGI (www.informatics.jax.org) provides gold-standard annotations to describe mouse models in the context of both the known alleles and strain backgrounds of the mice [1]. In MGI, phenotype and disease annotations are ascribed to a genetic representation (allele pairs and strain background) of the mice that displayed the phenotype. Sophisticated genetic engineering techniques have allowed for the production of multi-genic models with spatiotemporal control of gene expression and the introduction of multi-color reporters. These increasingly complex models may include both causative mutations and non-causative transgenic tools [2]. To relate phenotype and disease annotations made to a genotype in MGI with the gene, genomic marker, or transgene containing the causative mutation, non-causative markers, such as transgenic tools (e.g., recombinases and reporters), need to be computationally excluded from consideration. For example, mice carrying an inducible knock-in of a mutant form of mouse *Kcnj11* in the *Gt(ROSA)26Sor* locus and a transgene expressing *cre* recombinase in pancreatic cells, Tg(Ins2-cre)23Herr (genotype MGI:4430413), are annotated to the Mammalian Phenotype ontology (MP) [3] term ‘decreased insulin secretion’ (MP:0003059) and are a model of permanent neonatal diabetes mellitus (OMIM:606176) [4]. The phenotype and disease annotations are correctly associated with *Kcnj11*. However, the annotations should not be linked with the *cre* recombinase transgene or *Gt(ROSA)26Sor* since neither directly causes the phenotypes or disease displayed by the mice.

MGI is implementing improvements throughout the database to enhance the ability of users to evaluate the function of genes. As part of this, phenotype and disease associations at the level of the gene are now being presented (see below) in multiple locations in the MGI website. The gene-level associations give users an overview of the phenotypes and diseases associated with a gene that can be challenging to decipher from detailed model annotations. For both phenotypes and disease, creating a gene-level annotation implies that mutations in this gene cause the associated phenotype or disease. Therefore, the gene-level annotations may be useful to identify candidate genes for specific phenotypes and/or diseases. To create these gene-level associations, we have developed rules to algorithmically identify and computationally separate causative mutations from transgenic tools in complex mouse genotypes.

The first and simplest implementation of the rules excluded all complex genotypes and removed recombinase and wild-type alleles prior to inferring relationships. The need to separate causative mutations from transgene tools can best be illustrated by example. The complex genotype *Apoe* ^*tm1Unc*^/ *Apoe* ^*tm1Unc*^ *Fasl* ^*gld*^/*Fasl* ^*gld*^ on an inbred C57BL/6 strain genetic background (MGI:5514345) is annotated to the human disease Systemic Lupus Erythematosus, SLE (OMIM:152700) [5]. Inferring a causal relationship between *Apoe* and/or *Fasl* and SLE may or may not be correct, since it is unclear whether one or both genes are responsible for the observed phenotype. For complex genotypes such as this one, the algorithm does not derive any gene annotations. Conversely, *Smo* ^*tm1Amc*^/*Smo* ^*tm2Amc*^ *Isl1* ^*tm1(cre)Sev*^/*Isl1* ^*+*^ mice on a mixed 129 strain genetic background (MGI:3689403) are annotated to the phenotype ‘perinatal lethality’ (MP:0002081) [6]. The *Isl1* recombinase allele is present to drive deletion of the loxP-flanked *Smo* allele in the cardiovascular system; thus, we do not want to associate the perinatal lethality phenotype with *Isl1*. As we can clearly identify the non-causative allele and distill this genotype to alleles associated to a single gene, we derive a relationship between the phenotype ‘perinatal lethality’ and the gene *Smo*.

Other databases presenting phenotype and disease annotations for model organisms also have to decide when an annotation to a model can used to infer information about gene function. For example, the Zebrafish Model Organism Database (ZFIN, www.zfin.org, [7]) annotates phenotypes to a fish line that includes the alleles, transgenes and/or morpholinos used in an experimental cohort. Each allele and morpholino has an asserted relationship to a gene. Gene level annotations are then inferred for lines where only 1 asserted gene relationship exists (Y. Bradford, personal communication). Gene level annotations are not inferred for fish with more than one asserted gene relationship or for fish expressing non-reporter transgenes. This is similar to the early stages of the MGI algorithm. A key difference between mouse and zebrafish models, for the purpose of inferring gene annotations, is the widespread use of knock-in mutations in mouse where asserting the gene to allele relationship is less straightforward.

In contrast to the restrictive approach taken by ZFIN and MGI, the Monarch Initiative (monarchinitiative.org, [8]), which integrates data from both MGI and ZFIN as well as many other sources, infers gene annotations for all genes in a model. Thus, in the example above (*Apoe* ^*tm1Unc*^/ *Apoe* ^*tm1Unc*^ *Fasl* ^*gld*^/*Fasl* ^*gld*^) gene annotations would be inferred for both *Apoe* and *Fasl* (M. Brush, personal communication). This approach maximizes the number of gene-to-phenotype annotations but means the user will need to evaluate the results to remove false positive associations.

In the current implementation, presented below, the algorithm we have developed excludes additional transgenic tools, accounts for the introduction of expressed genes in alleles, and deals with multi-genic mutations. This approach increases the number of derived gene annotations, while attempting to reduce both the number of false positive and false negative annotations. While the precise implementation would not be of use to other databases the logic behind the algorithm should be transferable.

## Gene annotation derivation rules

Refinement of the derivation rules to eliminate additional types of transgenic tools has been an iterative process. Various changes to the MGI database schema have facilitated the identification and removal of many types of transgenic tools and non-causative marker associations. Throughout this process we have worked to minimize the number of false positive associations. The overall goal of these rules is to eliminate transgenic tools alleles and then infer gene, multi-genic marker, or transgene relationships from genotypes with only a single remaining associated locus. Genotypes with multiple associated loci are not used to infer gene relationships, with a few exceptions (see below). Recent re-implementation of these rules in a consistent manner across all MGI products has improved the gene annotation data quality at the display level and allowed us to make this data set available for export.

### Details of the annotation derivation rules

**Fig. 1:**
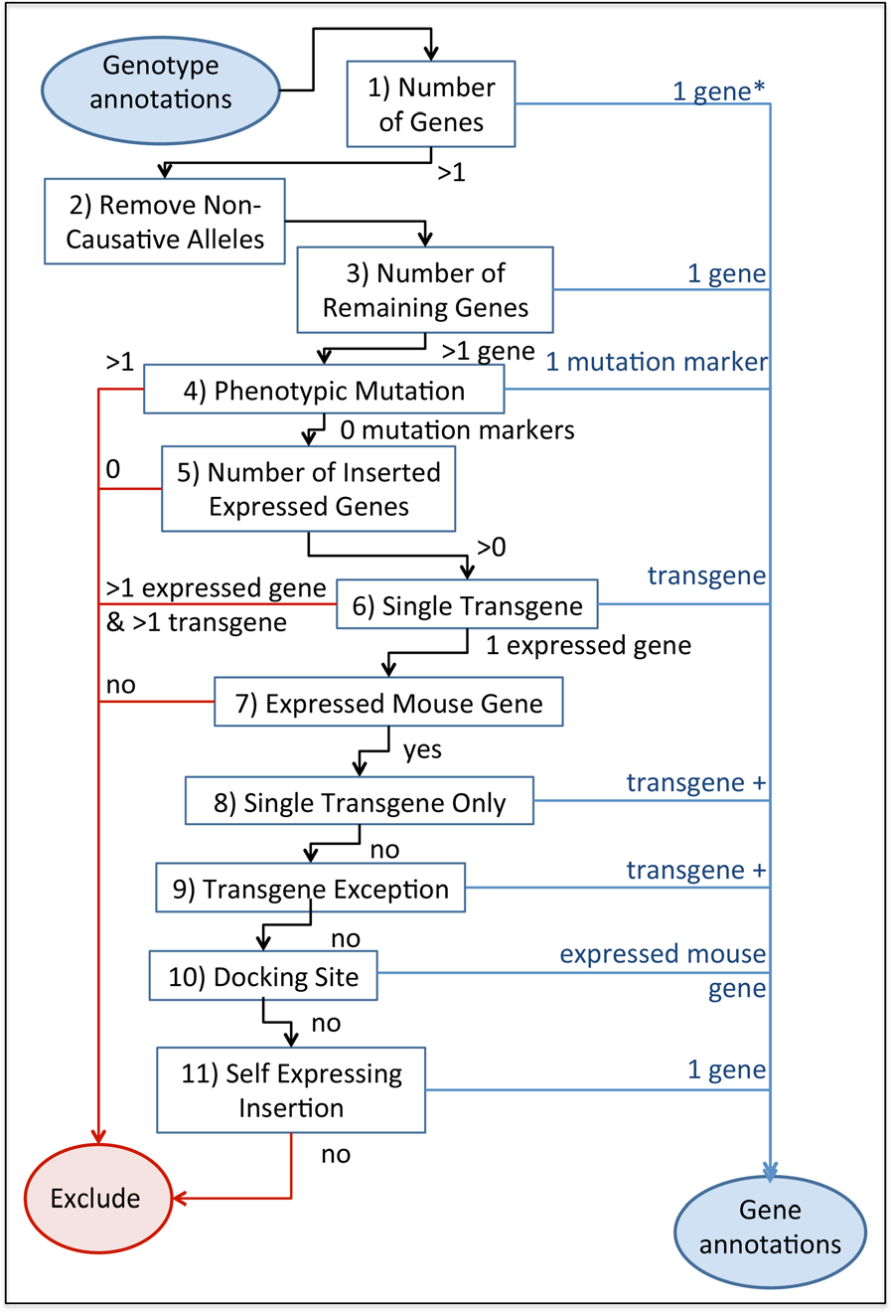
Flow chart for the application of gene annotation derivation rules. One gene*, annotations are derived only for certain cases of genotypes containing a single gene. See text for additional details. Transgene+, gene annotations are made to the transgene and an endogenous mouse gene

In the application of the derivation rules, genotypes are processed in a step-by-step fashion (see Fig. 1). First, the number of genetic loci associated with all alleles in the genotype is determined (Fig. 1, box 1). Genetic loci include: genes within the mutation region, genes expressed by the allele, transgene markers, and phenotypic markers. For example, the alleles *App* ^*tm1Dbo*^, Tg(tetO-Notch4*)1Rwng, and Del(7Coro1a-Spn)1Dolm (MGI:2136847, MGI:4431198, MGI:5569506 respectively) are associated with one, two, and forty loci, respectively. The two loci associated with Tg(tetO-Notch4*)1Rwng are the transgene itself and the expressed mouse gene, *Notch4*. The forty loci associated with Del(7Coro1a-Spn)1Dolm include the deletion region itself (recorded in MGI as a single, unique genetic marker) and all thirty nine endogenous mouse genes overlapping the deletion region. Gene-to-phenotype and gene-to-disease annotations can then be derived for the genes in nearly all genotypes with a single associated genetic locus (see docking sites below for the exception).

For genotypes including more than one locus, such as those described above, non-causative alleles are identified and computationally excluded from consideration. Non-causative allele types in the algorithm include: transgenic transactivator alleles, transgenic reporter alleles, knock-in and transgenic recombinase alleles, and wild-type alleles. Since many knock-in transactivator and reporter alleles may also be knock-out alleles that are causative for a phenotype, only transgenic alleles of these types are excluded. For recombinase alleles, curation in MGI distinguishes between conditional genotypes, where these alleles function as a recombinase, and non-conditional genotypes, where these alleles may be causative; therefore, both transgenic and knock-in recombinase alleles may be eliminated when the genotype is conditional. When the genotype is not conditional, recombinase alleles are retained. For a recombinase or transactivator allele to be excluded, it must express only a single gene. In cases where another gene is expressed, the allele is retained. For example the recombinase allele Tg(Tyr-cre/ERT2)1Lru (MGI:3617509) is excluded at this stage, so no derived annotation to the transgene is computed as a result of this allele. But the allele Tg(Tyr-cre/ERT,-Hras1*,-Trap1a)10BJvde (MGI:4354013) is retained, as it expresses both *Hras1* and *Trap1a* in addition to *cre*. Additional rules described below address whether and how to derive annotations to those genes. Motifs (ERT2, ERT) designed to alter the expression of *cre* are not curated as expressed genes and are therefore ignored by the algorithm.

After excluding non-causative alleles, the number of remaining loci is determined for each genotype. Gene-to-phenotype and gene-to-disease annotations are then derived for genes and genomic markers in genotypes with a single remaining locus. For genotypes with more than one remaining locus, further processing is done to identify additional cases where gene annotations can be derived. If the genotype is associated with a single multi-genic marker (e.g., Del(7Coro1a-Spn)1Dolm) and one or more affected genes located in the region, then annotations are derived for the multi-genic marker and not for the individual endogenous genes in the region (Fig. 1, box 4). Genotypes associated with more than one multi-genic mutation or with a multi-genic marker and any markers outside the mutation region are excluded and annotations are not derived for any of the genes or genomic markers involved.

The number of inserted expressed genes is then considered. Inserted expressed genes are genes that have been introduced into the mouse genome and the gene product is expressed in one or more tissues of the mouse. Genotypes with multiple associated markers and no inserted expressed genes are eliminated. Genotypes associated with multiple inserted expressed genes are associated to the transgenic locus only, if there is a single transgene associated with the genotype and no additional endogenous genes (Fig. 1, box 6). In this case, it is assumed that the transgene is expressing all of the inserted expressed genes and that the transgene as a whole, not the individual expressed genes, is causative for the phenotypes or diseases annotated to the genotype. For these genotypes, transgene-to-phenotype and transgene-to-disease annotations are derived. Derived annotations are not created for the inserted expressed genes. Other genotypes having more than one inserted expressed gene are excluded and no gene or transgene annotations are derived.

Genotypes associated with only a single inserted expressed gene (Fig. 1, box 7) are divided into two types: those expressing a mouse gene and those expressing a non-mouse gene. Genotypes associated with an expressed non-mouse gene are eliminated. No assumption is made that the phenotypes or diseases displayed would also be produced if the orthologous mouse gene had been used instead. Gene-to-phenotype and gene-to-disease annotations may be derived for a transgene and also an endogenous mouse gene in two cases: 1) if the genotype contains only a single transgene which carries a single inserted expressed mouse gene (Fig. 1, box 8); 2) if the transgene, inserted expressed mouse gene, and the single endogenous gene that is the same as the inserted expressed mouse gene are associated with the genotype (Fig. 1, box 9). In both cases annotations are derived for both the endogenous mouse gene and the transgene (Fig. 1, “transgene + ”).

Three genes (*Gt(ROSA)26Sor*, *Col1a1*, *Hprt*) are commonly used, based on examination of alleles in MGI, as ‘docking sites’ in mouse to knock-in expressed genes, frequently under the control of a heterologous promoter sequence. For example, of the 63 alleles of *Col1a1* in MGI with the attribute “inserted expressed sequence”, 55 have a construct inserted in the untranslated region based on the molecular description in MGI (12/7/15). For genotypes associated with a docking site and a single expressed mouse gene, gene-to-phenotype and gene-to-disease annotations are derived for the expressed gene and not for the docking site. There are no known phenotypes or diseases ascribed to mutations in *Gt(ROSA)26Sor* (MGI:104735, [9]). Therefore, no derived annotations are created for *Gt(ROSA)26Sor*, even when there are no associated expressed genes in MGI. MGI currently only annotates expressed genes with an ortholog in mouse; therefore, not all *Gt(ROSA)26Sor* alleles with an inserted expressed gene have an associated expressed gene. For example the allele *Gt(ROSA)26Sor* ^*tm1(gp80,EGFP)Eces*^ (MGI:5004724) expresses a gene from the Kaposi sarcoma herpes virus that does not have an ortholog in mouse. The phenotypes displayed by mice carrying this allele are the result of expression of the viral gene but as there is no display in MGI for any gene-to-phenotype annotations for a viral gene with no mouse ortholog, no derived annotations are created. Insertions in *Col1a1* (MGI:88467) and *Hprt* (MGI:96217) are typically made without altering normal endogenous gene function. For *Col1a1* and *Hprt* alleles, annotations are derived for the inserted expressed gene when one is present. If no expressed genes are present then annotations are derived for the docking site gene itself (Fig. 1, box 10).

The final case where gene annotations are derived is when the inserted expressed mouse gene is identical to the endogenous gene (Fig. 1, box 11). No gene annotations are created for any remaining genotypes.

### Gene annotation derivation examples

**Fig. 2:**
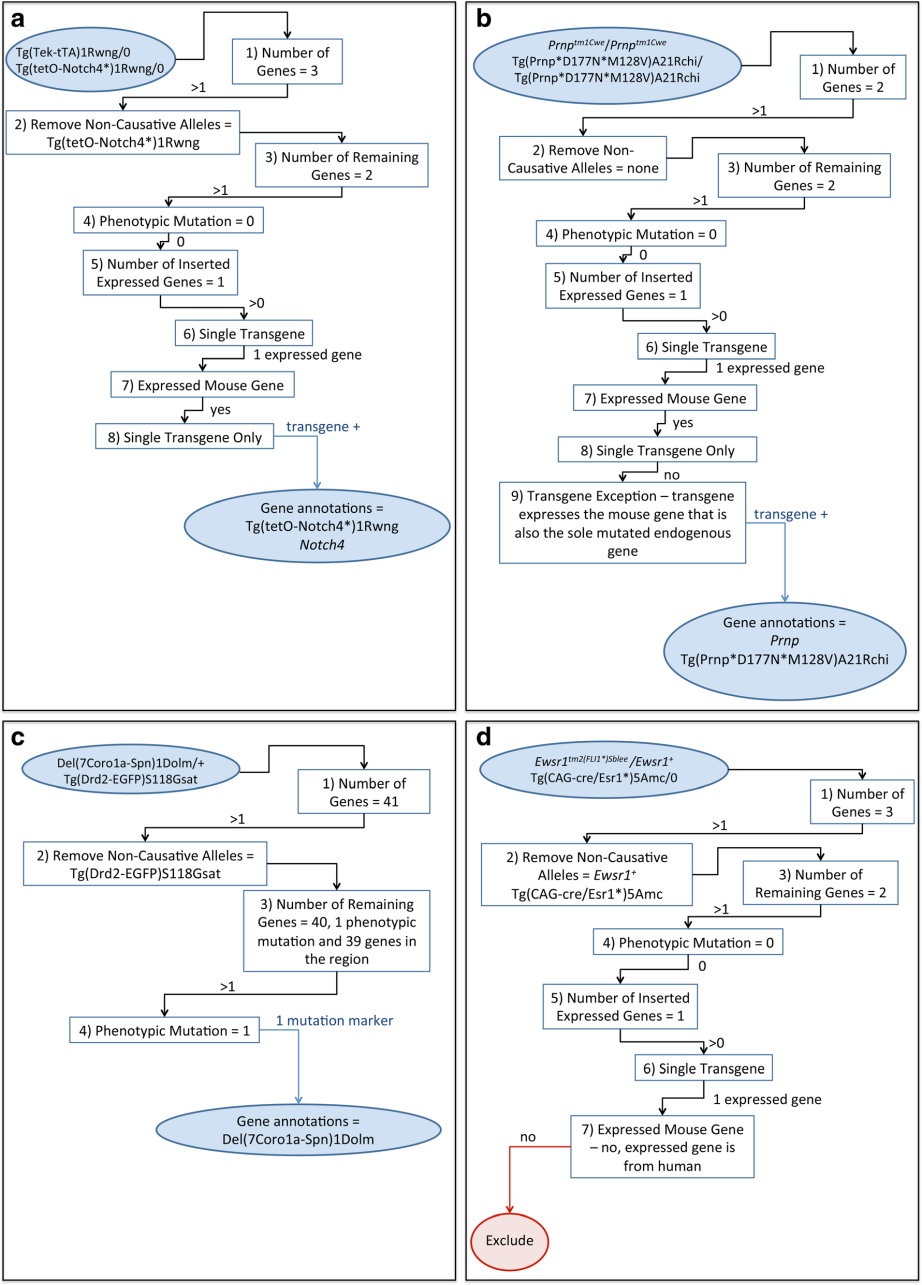
Overlay of specific genotype examples on the flow chart of the gene annotation derivation rules. **a** Processing of a genotype that results in annotations to a transgene and endogenous mouse gene. **b** Processing of a genotype that fits the transgene exception rule, where the transgene expresses a mouse gene and the same endogenous mouse gene is mutated in the mice. **c** Processing of a genotype with a reporter transgene and phenotypic mutation affecting multiple genes. **d** Processing of a conditional genotype where no gene annotations can be derived

To illustrate the function of the derivation algorithm, four example genotypes have been overlayed on the flow chart (Fig. 2). For mice hemizygous for Tg(tetO-Notch4*)1Rwng and Tg(Tek-tTA)1Rwng (genotype MGI:5502689, Fig. 2a), the transactivator expressing transgene Tg(Tek-tTA)1Rwng is excluded from consideration. This leaves 2 remaining genes, Tg(tetO-Notch4*)1Rwng and *Notch4*. As this leaves a single transgene marker and a single expressed mouse gene, gene level annotions are derived for both the transgene and the expressed mouse gene. For mice homozygous for *Prnp* ^*tm1Cwe*^ and Tg(Prnp*D177N*M128V)A21Rchi (genotype MGI:3836994, Fig. 2b) there are no non-causative alleles to remove. The single transgene in this case expresses the same mouse gene that is mutated by the allele *Prnp* ^*tm1Cwe*^ leaving the genotype associated with two genes, mouse *Prnp* and Tg(Prnp*D177N*M128V)A21Rchi. As this fits the requirements for the transgene exception (Fig. 2, box 9) annotations are derived for both the endogenous mouse gene and the transgene. For mice heterozygous for the deletion Del(7Coro1a-Spn)1Dolm and hemizygous for the reporter transgene Tg(Drd2-EGFP)S118Gsat (genotype MGI:5571091, Fig. 2c), the reporter transgene is excluded from consideration. As the deletion marker is associated with the 39 genes in the deletion region, this genotype falls into the Phenotypic mutation class for purposes of the algorithm. Gene annotations are derived for the deletion marker but not for the 39 genes in the deletion region (Fig. 2c, box 4). Mice heterozygous for *Ewsr1* ^tm2(FLI1*)Sblee^ and hemizygous for Tg(CAG-cre/Esr1*)5Amc (genotype MGI:4429149, Fig. 2d) illustrate a case where gene annotations are not derived. While two non-causative alleles are removed by the algorithm, the *cre* transgene and wild-type allele of *Ewsr1*, after processing is complete there are still two genes associated with the genotype, *Ewsr1* and *FLI1*. As the gene knocked into *Ewsr1* is not a mouse gene this genotyope is excluded at box 7 in the flow chart. Even if the expressed gene had been a mouse gene this genotype would have been excluded as the expressed gene is not the same as the mutated endogenous gene.

### Output of the rules

Once all genotypes with phenotype or disease annotations have been processed by the derivation rules the set of derived gene annotations are used throughout MGI, HMDC and MouseMine. As currently implemented, the rules result in derived gene-to-phenotype and gene-to-disease annotations for over 16,000 and 2200 mouse markers, respectively, starting from over 57,000 and 4800 genotypes with at least one phenotype and disease annotation, respectively (as of 1/4/2016). Of the over 57,000 genotypes processed, almost 40,000 contain only mutations in a single marker (Table 1). Gene level annotations could be derived from these genotypes using the simplest possible rule (only derive annotations when there is one marker associated with the genotype). Use of the derivation algorithm allows a further almost 8000 genotypes to be processed and marker level annotations created. This represents an almost 14 % increase in the number of genotypes contributing phenotype annotations at the marker level. Of the approximately 18,000 multiple marker genotypes, conditional genotypes and genotypes involving alleles expressing inserted genes are two important subsets. Conditional genotypes are primarily processed by removal of recombinase alleles. There are currently over 7000 genotypes where a recombinase allele is removed (Table 2). The ability to include special and temporal specific phenotypes in the gene level annotations enhances the overall picture of gene function MGI provides to users. There are over 3700 alleles (knock-in and transgenes) expressing at least one inserted sequence involved in nearly 4800 genotypes currently in MGI (as of 12/28/15). Over 2000 of these alleles express a mouse gene and may therefore potentially contribute to gene level annotations. Incorporation of these overexpression and misexpression induced phenotypes improves both the overall picture of gene function and the relation of mouse models of human disease to genes.

**Table 1:** Number of genotype and gene annotations processed by the derivation algorithm

Genotypes with MP and/or OMIM annotations	Number of genotypes (percent of total, 1/4/2016)	Number of genes
Total	57,920	
With Derived Gene Annotations	47,869 (82.6 %)	16,044
One Marker Genotypes (Fig. 1, box 1)	39,873 (68.9 %)	14,074
Resolved Multiple Marker Genotypes	7996 (13.8 %)	3870 (1970 novel markers)

**Table 2:** Breakdown of resolved multiple marker genotypes. These numbers only include genotypes with MP or OMIM annotations that have more than 1 marker

Non-causative alleles in genotypes	Number of genotypes processed (as of 1/4/2016)	Number Alleles
Recombinase alleles^a^	7,015	936
Reporter transgenes	256	157
Transactivator transgenes	282	84
Wild-type alleles	5,371	1,577

^a^Only counting recombinase alleles in conditional genotypes which have MP/OMIM annotations

There is a potential for the creation of false positive and false negative annotations by the derivation algorithm. One possible source of false positive annotations is the use of expressed gene relationships to identify when an allele is expressing a transcript that may alter the phenotype. For example, the gene *Col1a1* has 64 targeted alleles with the attribute “inserted expressed sequence” of these 58 have an association to an expressed gene. Of the remaining 6 alleles, 5 are alleles where an interfering RNA (RNAi) has been inserted into the gene. Determining how to represent the relationship between an RNAi expressing allele and the gene targeted by the RNAi is one of MGI’s future projects. During the development of the algorithm the use of the “inserted expressed sequence” attribute was still in development so the presence of an association to an expressed gene was used. We are reviewing the possibility of changing the algorithm to use the presence of the “inserted expressed attribute” instead of the presence of an expressed gene association, as this would improve our handling of these cases.

One possible source of false negative annotations is the limitation of “docking site” alleles to only *Col1a1*, *Hprt* and *Gt(ROSA)26Sor*. For example, annotations from the genotype MGI:5544092 could be associated with the mouse gene *Edn2* if the marker for the intergenic insertion site in the allele *Igs1* ^*tm11(CAG-Bgeo,-Edn2)Nat*^ was excluded from consideration. Instead of expanding the list of markers used for docking sites, we are exploring implementation of a “Docking Site” attribute that could be applied to specific alleles. This would avoid the need to modify the algorithm when new docking sites are encountered but would require back annotation of existing alleles. Another source of false negative annotations is the use of reporter genes that are a mouse gene or with an ortholog in mouse. For example, there are 63 knock-in alleles that use the mouse gene *Tyr* as a coat color reporter. Other than the pigmentation phenotype, phenotypes in these mice are the result of the mutated endogenous locus and not due to the expression of *Tyr*. However, using the current algorithm gene annotations are not derived for any of the annotated phenotypes. Correcting these would require modifying the algorithm to both ignore *Tyr* and teasing apart the phenotypes due to the reporter from those due to the mutated endogenous locus.

### Impact of MGI improvements

The development of these rules has relied heavily on the implementation of other database improvements in MGI. For example, the introduction of allele attributes allowed a distinction to be made between reporter transgenes that express only a reporter and transgenes that express a reporter and some other gene. The attributes were introduced as part of a restructuring of allele types into generation method and attributes. Attributes include both changes to the endogenous gene function (null/knockout, hypomorph) and characteristics of the inserted sequence (reporter, recombinase). Some attributes may apply to either the endogenous gene or the inserted sequence (hypomorph, modified isoform). An allele may have zero to many attributes but only one generation method. Certain attributes were then incorporated into the rules. These attributes include: reporter, recombinase, transactivator, and inserted expressed sequence. For example, exclusion of a reporter transgene requires the allele to have the generation method “transgenic” and the attribute “reporter” but not the attribute “inserted expressed sequence”. Therefore, the reporter transgene Tg(Cspg4-DsRed.T1)1Akik (MGI:3796063) that has only the attribute “reporter” is excluded as a non-causative allele. However, the reporter transgene Tg(CAG-Bmpr1a*,-lacZ)1Nobs (MGI:5473821) has multiple attributes including “reporter” and “inserted expressed sequence” and is retained.

The recent introduction of formalized data associations between transgenic and knock-in alleles and the genes expressed by these alleles has also been incorporated into the rules. MGI now annotates alleles expressing either a mouse gene or gene with a mouse ortholog to the gene being expressed. Alleles expressing inserted genes are then displayed on both the detail page for the endogenous locus where the insertion occurred and on the detail page for the mouse gene or mouse ortholog of the inserted gene being expressed. The rules make use of these associations to avoid assigning phenotypes to the endogenous gene in cases where an inserted expressed gene may be causative. They also allow annotations for phenotypes and diseases caused by transgenes expressing a mouse gene to be derived for the expressed mouse gene. For example, phenotypes for the knock-in allele *Ctnnb1* ^*tm1(Nfkbia)Rsu*^ (MGI:3039783) may be the result of loss of expression of *Ctnnb1* or the expression of *Nfkbia* and therefore no derived annotations are created. However, phenotype and disease annotations for the transgene Tg(Prnp*D177N*M128V)A21Rchi (MGI:3836986) are assumed to be the result of the expression of the mouse *Prnp* gene and derived annotations may be created for both the transgene and the expressed mouse gene.

### Use of the derived annotations in MGI

Implementation of the annotation derivation rules described here has improved both searching and display of gene-to-phenotype and gene-to-disease annotations in MGI. Gene level annotations are used on multiple displays and by multiple search tools in MGI. These displays and tools provide users with different ways to access, group, and filter the data. Regardless of how the user accesses the data, consistent results sets are now returned when searching for genes by a phenotype or disease.

**Fig. 3:**
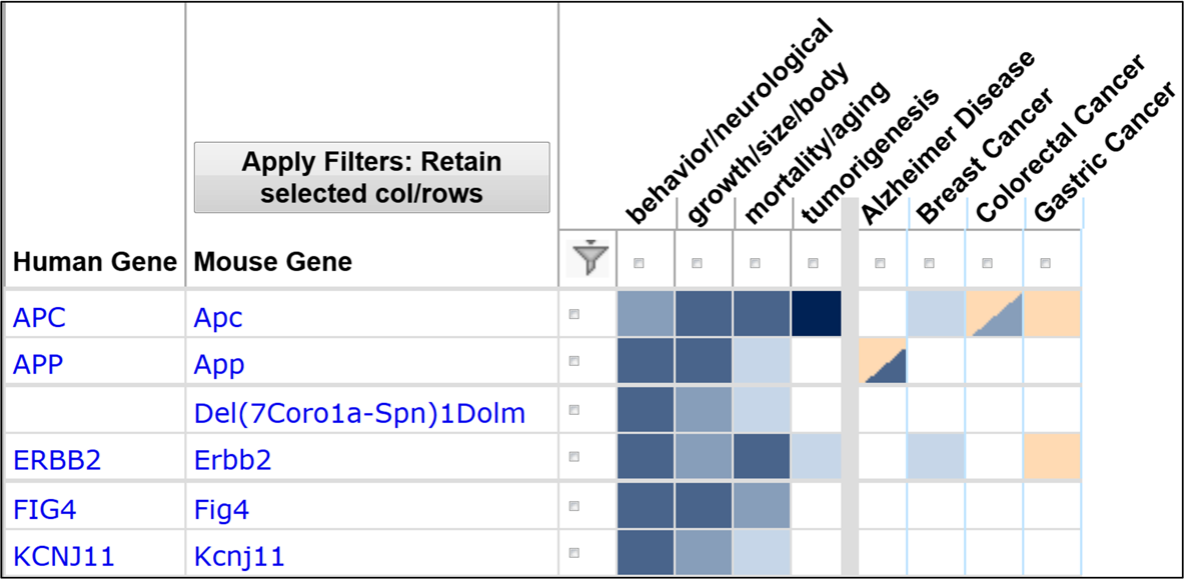
Display of derived gene-to-phenotype and gene-to-human disease annotations in the HMDC. A search was done for the genes *Apc*, *App*, *Erbb2*, *Fig4* and *Kcnj11*. Each row shows the derived gene-to-phenotype and gene-to-disease annotations for a mouse gene (*in blue*). Direct annotations of human genes to disease (*in orange*) are shown in the same row as the homologous mouse gene. Results have been filtered to reduce the number of rows and columns

**Fig. 4:**
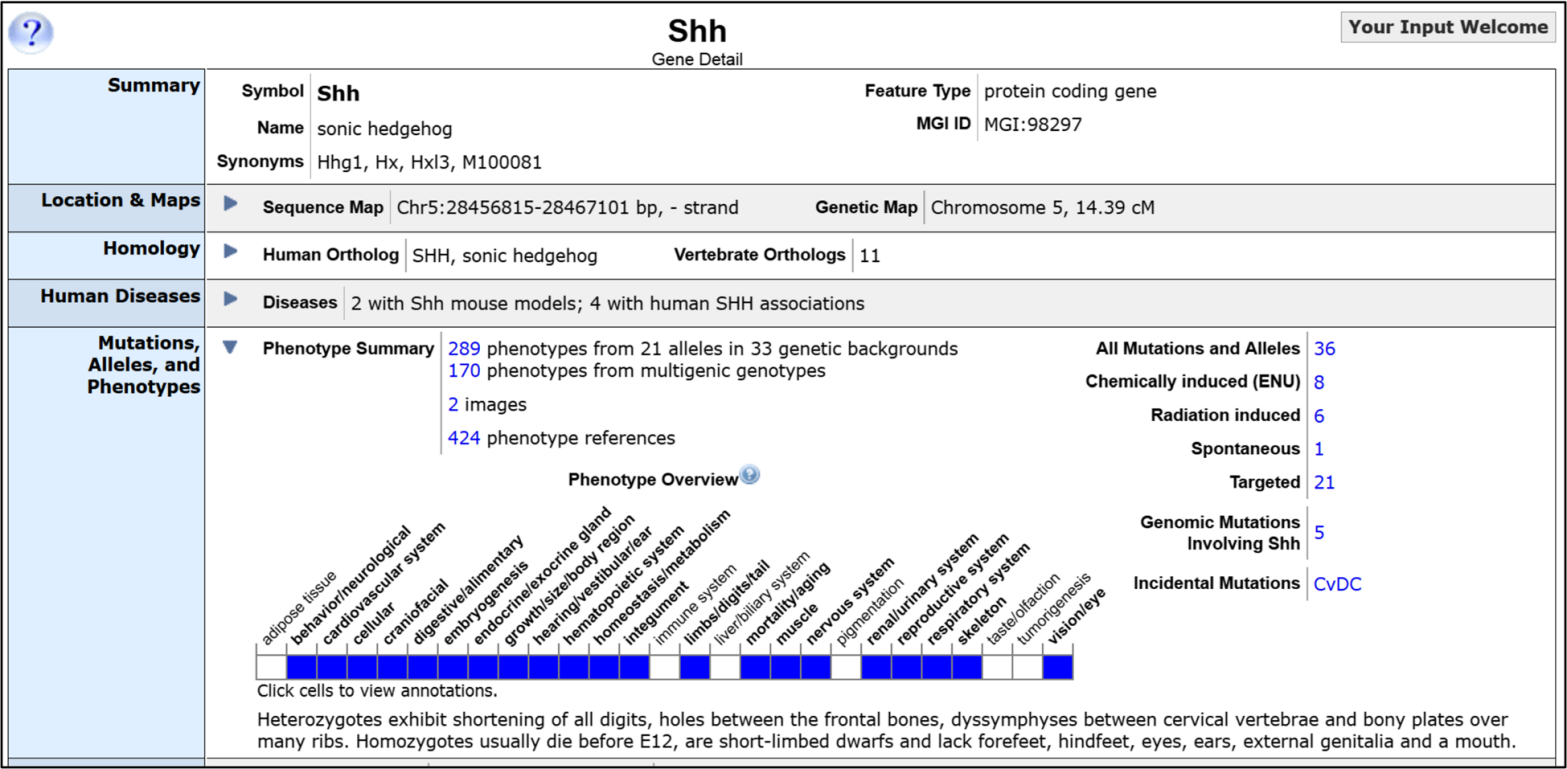
Display of derived gene-to-phenotype annotations on the *Shh* gene detail page in MGI. All Mammalian Phenotype system-level terms are shown. Blue boxes indicate abnormal phenotypes have been reported for that system. Blank boxes indicate absence of data for *Shh* mutants in that system in MGI

One way a user may access the derived annotations for a gene or set of genes is using the Human-Mouse: Disease Connection (HMDC, www.diseasemodels.org, Fig. 3). In the HMDC, searches for mouse data are restricted to only the derived gene-to-phenotype and gene-to-disease annotations. In the results, users may also access the set of genotype annotations used to generate the gene annotations, but multi-genic genotypes are excluded from the display. In MGI, the display of a mouse gene on a disease detail page is based both on the derived gene-to-disease annotations and on orthology relationships to known human disease genes. A gene that has both a derived gene-to-disease annotation and is orthologous to a known human disease gene is displayed in the human and mouse section of the page. Those without an orthology relationship but with a derived annotation are shown in the mouse only section. A similar division is made on the all models page for a disease, with multi-genic models that have neither gene orthologs nor derived annotations shown in the additional complex models section. The derived gene annotations are also incorporated into the updated design of the MGI gene detail page. With this modification, users see a summary graphic of the types of phenotypes caused by mutations in the gene (Fig. 4). On both the gene detail page and in the HMDC, gene level annotations are shown at the MP system level. Users may click through to see the detailed MP terms and associated allele pairs. This avoids the problem of displaying conflicting phenotypes (i.e., increased vs decreased body weight) at the gene level. From both locations users can access details and references to follow up on annotations of interest.

The Genes & Markers Query Form uses the derived annotations when a user searches by phenotype or disease to determine the set of genes and markers returned. The Batch Query tool uses the derived annotations to determine the set of phenotype terms returned for a gene. In this case, unlike in the HMDC, the details link includes both the genotypes used to derive the annotations and complex genotypes annotated to the same term or to a subclass of that term. The Gene Expression Database (GXD) Query Form uses the derived annotations to define a set of genes associated with a phenotype or disease. Users can then retrieve expression data for the genes in the set. MGI FTP reports for gene-to-phenotype and gene-to-disease associations (HMD_HumanPhenotype.rpt and MGI_OMIM.rpt) include only the derived annotations. Finally, MouseMine (www.mousemine.org [10]) makes use of the same set of rules and allows users to trace back to the alleles and genotypes underlying the derived annotation set. The connection to the source alleles allows users to filter the phenotypes based on allele attributes to find, for example, phenotypes for a gene caused by null mutations.

Other searches in MGI, such as the Quick Search and Phenotypes, Alleles & Disease Models Search, return the set of alleles for a phenotype or disease term and include annotations for both single- and multi-genic genotypes. Since these queries return alleles rather than genes, the rules for the derived annotations are not applied.

The return and display of gene-to-phenotype and gene-to-disease annotations are critical to evaluation and comparison of genes and disease models. In the HMDC, the gene level annotations allow users to refine a set of genes based on the phenotypes or diseases resulting from mutations in the gene before delving into the specifics of the models. On a disease detail page, users can identify disease models associated with mouse genes that are orthologous to known human disease genes and those that are not. The latter class provides a valuable source of potential new candidate human disease genes. With the Batch Query tool, a user can retrieve all phenotypes and diseases associated with a gene that can be exported for further analysis. The summary graphic on the gene detail page will allow users to rapidly review and compare the phenotype profiles of genes.

## Discussion

The use of rules to derive annotations has two major advantages over direct curation. First is the hands-on curatorial time-savings benefit. Curators need to enter only the genotype-to-phenotype or genotype-to-disease annotations and do not need to also annotate the gene relationships. Given the large number of existing annotations and the ongoing need to focus curation efforts to newly published literature, the elimination of the requirement for manual curation of gene relationships is vital. Second, using the rules insures consistency of annotation. While we strive for inter-curator consistency at MGI, some variability is inevitable. With the use of unified rules, the derived annotations are always consistent.

Despite the advantages of the derived annotation rules, a limitation of the use of rules to derive annotations as opposed to direct curation of these relationships is the loss of some potential annotations. One way annotations may be lost is due to failure to exclude non-causative alleles. For example, knock-in transactivator alleles cannot currently be excluded. Thus, no derived annotations can be made for mice with the genotype *Foxg1* ^*tm1(tTA)Lai*^/*Foxg1* ^*+*^, Tg(tetO-Gsx2,-EGFP)1Kcam/0 (MGI:4412090). Further, cases where a reporter gene is a mouse gene or has an ortholog in mouse (e.g., mouse *Tyr*, human *ALPP*) are captured in the count of expressed genes, but rarely do these genes contribute to a disease phenotype, when one is displayed. With modifications to MGI annotations and additional refinements to the rules we may be able to eliminate more of these allele types from gene relationship consideration, through automated processing.

The use of these rules currently also limits the derived annotations to only those caused by a single gene. The inclusion of disease and phenotype annotations that rely on the presence of mutations in multiple genes are completely excluded by the current algorithm. So gene-to-phenotype annotations are not created for either gene based on annotations for mice homozygous for both *Epn1* ^*tm1Ocr*^ and *Epn2* ^*tm1Ocr*^ (MGI:4356019), where the phenotypes are the result of combined loss of both genes and loss of either gene alone does not produce an abnormal phenotype[11]. While it would be possible in such a case to ascribe all phenotypes from the double homozygote to both genes, the situation is frequently more complex. In many cases, only some of the phenotypes displayed are caused by the double mutation while others are caused by only one of the mutations. Thus, decisions may need to be made at the individual Mammalian Phenotype term annotation level and not at the level of the genotype. In addition the potential for differences in strain background and annotation depth between genotypes to create false positive associations is increased relative to annotations inferred for genotypes with a single causative gene. For example, a subsequent paper looking at the impact of loss of expression of both *Epn1* and *Epn2* in the vasculature on tumor development [12] did not include either single homozygote as a control making it difficult to determine conclusively that loss of both genes is required for the phenotype. Similarly, mice homozygous for mutations in both *Cd80* and *Cd86* (MGI:3620124) have been reported to be a model for Insulin-Dependent Diabetes Mellitus (OMIM:222100) but single homozygotes were not examined and the strain background is different from that reported previously for the single homozygotes [13]. In this case, it is likely the mutations in *Cd80* and *Cd86* modify the disease phenotype but do not cause the disease as the mutations were moved into a strain (NOD) known to develop diabetes. Due to these issues and questions of how to distinguish multi-genic from monogenic phenotypes in the web display, attempting to distinguish between causal mutations, modifying mutations and annotation gaps for multi-genic genotypes was determined to be beyond the scope of the current algorithm.

Clarity of display also drove the decision to infer only gene-to-phenotype and gene-to-disease annotations for expressed mouse genes and not for expressed orthologs of mouse genes. Inferring a gene-to-disease relationship to the mouse gene for phenotypes in mice heterozygous for *Col1a1* ^tm1(CAG-IDH2*R140Q)Kkw^ (MGI:5582197) [14] would have resulted in the display of the mouse gene *Idh2* on the disease detail page for D-2-Hydroxyglutaric Aciduria 2 (OMIM:613657), giving the impression that the mouse gene has been used to model the disease when it is the human gene being expressed. However, since the species of the ortholog is currently stored in the database, future implementations of the MGI disease displays could use this information by, for example, providing links to humanized mouse models of a disease.

Another focus for improvement of the algorithm is the reduction of the number of remaining false-positive derived annotations. One source of false positives is genotypes where the strain background is responsible for the phenotype or disease displayed. In Mora et al. [15], mice homozygous for *Sell* ^*tm1Flv*^ on a congenic NOD background (MGI:3039435) were generated to investigate the effect of loss of *Sell* expression on insulin dependent diabetes (OMIM:222100). These mice show the same diabetic phenotype as wild-type NOD controls. However, the rules derive an annotation of *Sell* to diabetes based on the annotation of this genotype to this OMIM term. Refinements to MGI annotations and incorporation of strain background information into the derivation rules may allow us to exclude these genes from the results sets in the future.

## Conclusion

The conversion of gene-to-phenotype and gene-to-disease relationships in MGI from several variable rules used only for web page display to a single set of well-defined rules used to create derived annotations in the database improves both the consistency and accessibility of these relationships, as well as facilitates easier modifications to the rules. The derived gene-to-phenotype and gene-to-disease annotations are used for web display, downloads, and public reports and are available for export. Consumers of the exported data need to be aware of the restrictions placed on the annotations by the algorithm as this may alter interpretations of the data. Changes made to the rules can be seen throughout the database after any data update. The increased adaptability of these rules will aid our ability to keep pace with the changes in transgenic technology in the future.

## Abbreviations

HMDC: Human-Mouse: Disease Connection
MGI: Mouse Genome Informatics
MP: Mammalian Phenotype ontology
OMIM: Online Mendelian Inheritance in Man
